# An Improved Parameter Extraction Optimization Algorithm for RF Devices

**DOI:** 10.3390/mi16040432

**Published:** 2025-04-02

**Authors:** Shengsen Yang, Zihan Xu, Kun Ren

**Affiliations:** Innovation Center for Electronic Design Automation Technology, Hangzhou Dianzi University, Hangzhou 310018, China

**Keywords:** RF devices, parameter extraction, deterministic optimization algorithm, Ka-band filter

## Abstract

This paper proposes an improved parameter extraction optimization algorithm for radio frequency (RF) devices. The algorithm integrates parameter classification and correction, gradient-based performance handling, bias-aware updates, and group-based optimization strategies, achieving enhanced optimization accuracy, accelerated convergence, and improved stability. It effectively addresses the limitations of deterministic algorithms in RF device parameter extraction optimization, such as low efficiency, sensitivity to initial values, and unstable convergence. To validate the algorithm’s effectiveness, a Ka-band filter performance curve fitting case study was conducted. By comparing simulated curves with optimized fitted curves, the advantages of the algorithm in terms of optimization efficiency, accuracy, and convergence stability were demonstrated. Experimental results show that, compared to traditional optimization algorithms, the proposed method significantly improves curve fitting accuracy, computational efficiency, and stability, highlighting its application value in RF device parameter extraction.

## 1. Introduction

In recent years, with the continuous development and innovation in the field of RF circuit design, accurate RF device modeling has become a key factor in ensuring circuit performance. The accuracy of the device model not only directly affects the simulation precision of the circuit but also has a profound impact on design optimization efficiency and the performance reliability of the final product. However, traditional manual parameter adjustment methods are increasingly unable to meet the demands of emerging applications, especially when faced with complex design tasks and high precision requirements. To address this challenge, automated methods based on parameter optimization algorithms have emerged and gradually become important tools for RF device modeling and performance enhancement. Automated parameter optimization algorithms, by systematically adjusting design parameters, effectively compensate for the shortcomings of manual tuning, significantly improving design efficiency and optimizing device performance, especially in high-frequency complex scenarios, where they show significant value. Therefore, the development of efficient and accurate optimization algorithms to enhance the quality of RF device modeling and parameter extraction efficiency has become a critical research direction in the current RF technology field.

Based on the implementation strategies of optimization algorithms, RF device parameter extraction optimization methods can generally be divided into two categories: stochastic algorithms and deterministic algorithms. Stochastic algorithms, inspired by natural phenomena, such as Particle Swarm Optimization (PSO) [1] and Evolutionary Algorithms (EAs) [2], exhibit strong global search capabilities in parameter optimization through random mutation mechanisms. In contrast, deterministic optimization algorithms seek the optimal solution by solving specific mathematical functions and equations, with the entire optimization process being independent of randomness [3]. In actual RF device parameter extraction, the advantage of deterministic optimization algorithms lies in their result stability and consistency, meaning the same solution can be obtained in every experiment. This characteristic allows engineers to efficiently find the parameter combinations that meet performance requirements when constructing RF device models.

The parameter optimization process in RF device parameter extraction typically involves accurately establishing an equivalent circuit model and solving it based on specific design goals and constraints. However, the complexity of the model and the nonlinear characteristics of the physical constraints often pose significant challenges during the optimization process. In this paper, a modified optimization algorithm is proposed to address the limitations of existing RF device parameter extraction algorithms, aiming to improve the efficiency and effectiveness of model parameter extraction. The improved algorithm is capable of quickly finding local optima while maintaining the ability to converge towards the global optimum. Finally, the algorithm is compared with other optimization algorithms through the practical application of a filter design model, verifying its superiority in terms of optimization efficiency and precision.

## 2. Deterministic Parameter Optimization

The deterministic sizing design was initially proposed in [4], with the goal of optimizing the device dimensions in circuits with fixed biasing. To enhance the applicability of this algorithm in the RF device parameter extraction scenario, this paper presents several improvements and optimizations. This section will first introduce the basic principles of the improved RF device parameter extraction optimization algorithm, followed by a detailed discussion of the improvements in terms of algorithm efficiency and effectiveness. The improvements include the classification and updating of design parameters, methods for processing performance gradients, and the bias-aware weighting scheme. These optimization strategies collectively form the core of the improved RF device parameter extraction optimization algorithm.

**Figure 1:**
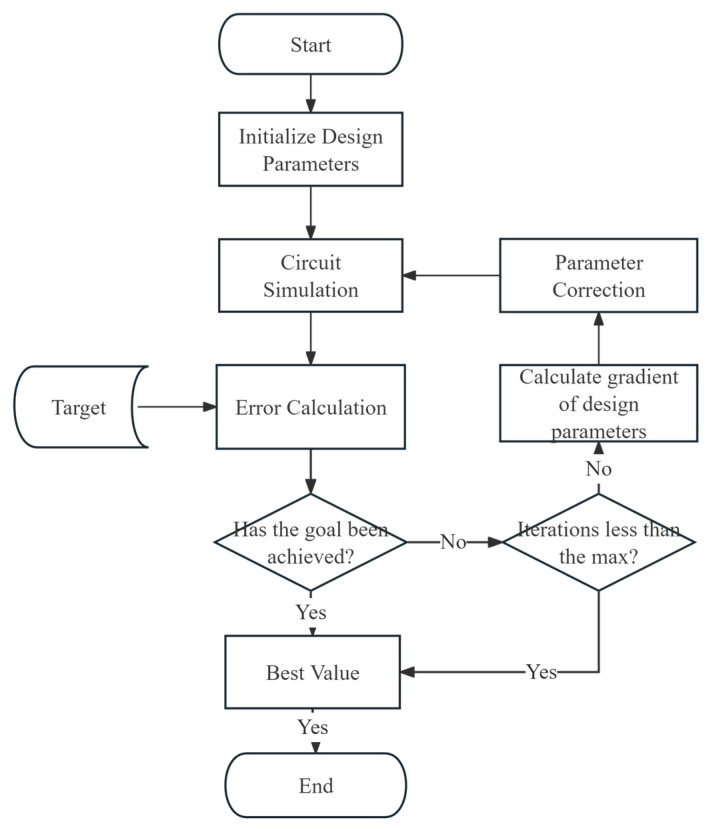
Flowchart of the RF device parameter optimization algorithm.

The improved algorithm is applied to the basic process of RF device parameter extraction, as shown in Figure 1. The specific steps include parameter initialization, parameter gradient calculation, parameter correction, and error calculation, among other key stages. Through step-by-step optimization strategies, this process significantly enhances the optimization efficiency and accuracy of the algorithm in complex models.

### 2.1. Parameter Classification and Correction in Algorithms

The core of the optimization of the RF device parameter extraction algorithm lies in the parameter correction at each iteration, which was initially implemented using the optimized Characteristic Boundary Curve (CBC) method [4].

First, the mathematical formulation of the RF device model is defined as follows:

(1) $$y=f\left(s\right)$$

where y represents the simulation output determined by the design parameters $s=\left[s_{1},s_{2},\ldots ,s_{n}\right]$, and $f\left(s\right)$ is a nonlinear function describing the RF device performance.

By linearizing the device performance at s, the performance gradient of the current design parameters s is computed as follows:

(2) $$g=\left(f\left(x+\Delta x\right)-f\left(x\right)\right)/\Delta x$$

Then, the CBC method is applied to the following objective function to compute the parameter correction x using g:

(3) $$\mathrm{minimize}:\sum \exp{\left(-\alpha \cdot E\left(z\right)\right)}^{2}+\lambda \cdot {\left\|z\right\|}^{2}$$

where the coefficient α is a constant used to adjust the parameter scale, and the variable λ controls the weight of the Euclidean squared regularization term for x. The error function $E\left(x\right)$ is defined as follows:

(4) $$E\left(z\right)=f\left(x\right)-f_{\mathrm{spec}}+g\cdot z$$

where $f_{spec}$ is the target error. Once x is computed, the design parameters are updated as follows:

(5) $$x_{\mathrm{new}}=x+z$$

The original CBC method was improved in [5], leading to the Generalized Boundary Curve (GBC) method, which employs a binary search technique to efficiently determine the parameter correction value. During the design parameter update process, some parameters may undergo significant changes while exhibiting small performance gradients, resulting in nonlinear parameter updates.

To address this issue, this paper further optimizes the GBC method by classifying design parameters according to their types, such as microstrip line length, width, and bias voltage. The correction for each type of design parameter is computed separately, and the improved error expression is formulated as follows:

(6) $$E\left(z_{i}\right)=f\left(x_{i}\right)-f_{\mathrm{spec}}+\left\|g_{i}\right\|\cdot z_{i}$$

where $x_{i}$ represents the i-th component of the parameter correction vector x, and $∥g_{i}∥$ denotes the performance gradient of the i-th design parameter. After computing $x_{i}$, the design parameters are updated according to Equation (5).

Although the improved parameter update algorithm requires additional computational resources, it provides more precise parameter corrections. Compared to the original CBC method, the improved GBC method demonstrates superior efficiency and adaptability in handling nonlinear problems. In particular, when dealing with highly nonlinear cost functions, the improved algorithm exhibits significantly enhanced stability and efficiency.

Moreover, since different types of design parameters exhibit varying sensitivities to parameter variations, computing the correction for each type of parameter separately—rather than applying a unified adjustment to all design parameters—ensures smoother and more precise updates during the iterative optimization process. Consequently, the proposed method outperforms the approach described in [5] in terms of both optimization efficiency and overall performance improvement.

### 2.2. Handling of Parameter Performance Gradient

Due to the varying sensitivity of RF device model performance to different design parameters, the performance gradients of some parameters may be significantly larger than those of others. These high-gradient parameters often dominate the value of $∥g_{j}∥$, which in turn leads to nonlinear behavior in the update process of low-gradient parameters, thereby affecting the stability and accuracy of the optimization process.

To address this issue, this paper proposes an improved gradient update algorithm, which preprocesses the performance gradients before computing parameter corrections. The specific steps of the preprocessing procedure include the following: (1) Gradient grouping: the gradients are classified into multiple gradient groups based on the types of design parameters. (2) Statistical analysis and filtering: within each gradient group, statistical analysis is performed to compute the average value of the other gradients in the group. If a particular gradient is significantly higher than the group average (e.g., exceeding five times the mean), it is removed from the gradient group. By implementing this preprocessing procedure, the influence of high-gradient parameters on the update process of other parameters is mitigated, ensuring smoother parameter updates. This approach not only enhances the robustness of gradient handling but also further improves the efficiency and accuracy of parameter selection.

### 2.3. Bias-Aware Update Scheme

Through multiple modeling experiments, it was observed that when the performance error of the device model is relatively large, adjustments to certain design parameters have a more significant impact on reducing the model error. Inspired by the dynamic weight allocation mechanism of the Ant Colony Optimization (ACO) algorithm [6], this paper proposes a bias-aware weighting scheme and integrates it into the improved optimization algorithm to further enhance optimization performance.

This scheme dynamically adjusts the weights of design parameters based on variations in model accuracy, enabling adaptive weight allocation. The specific weight calculation formula is as follows:

when using $f_{Lspec}$, the formula is as follows:

(7) $$\left\{\begin{array}{c} \varphi_{k\in B}=1,\varphi_{k\in O}=0;if\;f\left(x_{k}\right)\leq a*f_{\mathrm{Lspec}}\\ \varphi_{k\in B}=1-{\left(\frac{f_{\mathrm{Lspec}}}{f\left(x_{k}\right)}\right)}^{b},\varphi_{k\in O}={\left(\frac{f_{\mathrm{Lspec}}}{f\left(x_{k}\right)}\right)}^{b};else \end{array}\right.$$

when using $f_{Uspec}$, the formula is as follows:

(8) $$\left\{\begin{array}{c} \varphi_{k\in B}=1,\varphi_{k\in O}=0;if\;f\left(x_{k}\right)\geq a*f_{\mathrm{Uspec}}\\ \varphi_{k\in B}=1-{\left(\frac{f\left(x_{k}\right)}{f_{\mathrm{Uspec}}}\right)}^{b},\varphi_{k\in O}={\left(\frac{f\left(x_{k}\right)}{f_{\mathrm{Uspec}}}\right)}^{b};else \end{array}\right.$$

where $f_{Uspec}$ and $f_{Lspec}$ represent the upper and lower limits of model accuracy requirements, respectively. The parameter $a\in \left[0,1\right]$ is a weight control parameter used to balance the importance of the design parameter group and other parameter groups during the optimization process. When a is close to 1, the weight of the design parameter group dominates, and the optimization process tends to adjust the design parameters. Conversely, when a is close to 0, the optimization process tends to explore other parameter groups.

b is a natural number used to adjust the distribution characteristics of the weights, controlling the distribution of the weight factors. A larger b value concentrates the weight on a few parameter groups, while a smaller b value results in a more uniform weight distribution. These two parameters are critical hyperparameters for the group-based deterministic optimization algorithm. The weight $\varphi_{k\in B}$ and $\varphi_{k\in O}$ represent the weight factors of the design parameter group and the other parameter groups, respectively. Weight factors are typically set based on the sensitivity of different parameter groups to the optimization objective function. Parameter groups with higher sensitivity should be assigned greater weight to enhance optimization efficiency.

By incorporating the above bias-aware weighting scheme, the parameter update formula is modified as follows:

(9) $$s_{k,\mathrm{new}}=s_{k}+\varphi_{k}\cdot x_{k}$$

This scheme dynamically adjusts the correction magnitude of design parameters and other parameters by sensing deviations in model accuracy, thereby achieving a more efficient and precise optimization process. This bias-aware update mechanism, inspired by the core concept of the Ant Colony Optimization (ACO) dynamic weight allocation strategy, enables the proposed improved algorithm to effectively balance global search and local convergence performance, significantly enhancing optimization efficiency and the stability of optimization results.

### 2.4. Group-Based Optimization

Although the optimization algorithm proposed in [4] improves optimization efficiency to some extent, its exploration space is constrained by the initial design point, making it prone to local optima. To address this issue, this paper proposes an improved group-based exploration scheme, which enhances global optimization capability by expanding the search space. In addition to the original termination condition (10), a new termination criterion (11) is introduced to determine whether the optimization process has stagnated:

(10) $$f\left(x_{i}\right)\;\mathrm{meets}\;f_{\mathrm{spec}}$$

(11) $$f\left(x_{i+1}\right)\&f\left(x_{i+2}\right)\&\ldots \&f\left(x_{i+\tau }\right)\;\mathrm{are}\;\mathrm{not}\;\mathrm{better}\;\mathrm{than}\;f\left(x_{i}\right)\;\mathrm{by}\;\delta$$

where τ is a user-defined number of consecutive optimization iterations, and δ is the minimum performance improvement tolerance, serving as a criterion for detecting optimization stagnation. If the performance does not exceed the predefined threshold over τ consecutive iterations, the optimization process is deemed to have stagnated.

However, while condition (10) helps deterministic optimization escape local optima to some extent, its improvement remains limited due to the restricted exploration space imposed by the initial design point. To overcome this limitation, this paper proposes a group-based exploration scheme, which creates multiple deterministic optimization individuals, each starting from a randomly generated initial design point and performing independent optimization. This approach significantly expands the search space and prevents global optimization failure caused by the poor quality of a single initial design point.

The improved RF device parameter extraction optimization algorithm proposed in this paper is called IRDPO (Improved RF Device Parameter Optimization Algorithm). This algorithm integrates the key improvements introduced in Section 2.1, Section 2.2 and Section 2.3: parameter correction calculation, bias-aware design parameter update, and termination condition optimization. The specific process is shown in Algorithm 1, which includes parameter initialization, independent optimization, parameter update, termination judgment, and result integration.
**Algorithm 1**: Improved RF Device Parameter Optimization Algorithm
Initialize the Gro group including M individuals; Randomly generate M initial design points P; For each individual in Gro:    Start optimization with one of P;   While iteration index k ≤ maximum allowed number of iterations:       Evaluate the performance of Ind based on the current design parameter values $s_{k}$       Update the best performance of Gro(i.e., $G_{best}$);       If any Ind’s performance target value Spec:           Break While;       For each type (index of  k) of design parameters of Ind:           Calculate the gradient of parameters;           Calculate the correction of parameters;           Calculate the updating weight of parameters;           Update the value of parameters using $s_{k,i+1}=s_{k,i}+\varphi_{k}\cdot x_{k}$       End For   End While End For Return $G_{best}$ and its corresponding circuit parameter values $s^{*}$

In each iteration, IRDPO only needs to store the current parameter values and the objective function values. Each iteration primarily involves the calculation of the objective function and parameter update. If the time complexity of the objective function calculation is $O\left(f\left(n\right)\right)$, and the number of parameters is d, then the time complexity of a single iteration of the improved algorithm is $O\left(d\cdot f\left(n\right)\right)$. Compared to random algorithms, which are influenced by population size, IRDPO has a lower time complexity. At the same time, IRDPO only needs to store the current parameter values and objective function values in each iteration, resulting in a space complexity of $O\left(d\right)$. In contrast, PSO and the Genetic Algorithm (GA) need to store the entire population’s information, resulting in a space complexity of $O\left(p\cdot d\right)$.

## 3. Experimental Case Study

The Ka-band filter is an efficient radio frequency (RF) signal processing device with a working frequency range of 20 GHz to 40 GHz. In wireless communication or radar systems, the Ka-band filter serves as a signal processing unit, selectively transmitting target frequency band signals and suppressing interference signals, making it a typical parameterized RF functional device. Therefore, this paper selects the parameter optimization of the Ka-band filter as a case study to validate the improved RF device parameter extraction optimization algorithm. The filter utilizes advanced microwave design technology, offering good frequency band selectivity and low insertion loss, effectively suppressing unnecessary frequency band interference, thereby improving signal quality [7].

Typically, each set of microstrip lines corresponds to a resonant unit. Resonators are key components in RF circuits, widely used in filters, oscillators, and other RF devices. The basic principle relies on the self-resonance characteristic of the circuit, generating resonance within a specific frequency range. Common resonator structures include microstrip resonators, cavity resonators, and dielectric resonators. A microstrip resonator is generally composed of a microstrip line and a ground layer, with the resonant frequency controlled by adjusting the microstrip line’s geometric dimensions. The length of the microstrip line determines the resonant frequency, and variations in length directly affect the frequency’s increase or decrease. Additionally, the microstrip resonator can achieve high selectivity and low insertion loss through its structural design, enabling effective signal selection and filtering across multiple frequency bands. Therefore, a rational resonator structure design is crucial to the performance of the filter [8,9].

**Figure 2:**
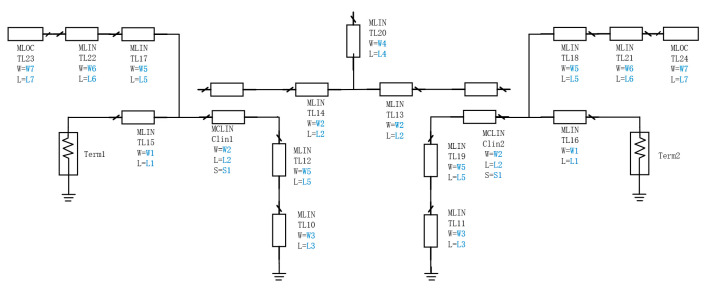
Schematic diagram of the Ka-band filter.

Based on the symmetrical structure of the filter, this case study identifies 15 design parameters, including the lengths (L), widths (W) of the microstrip lines, and the spacing (S) of the coupled microstrip lines. By optimizing these parameters, the filter’s key performance indicators, such as the insertion loss and reflection coefficient, can be effectively controlled, enabling accurate modeling of the filter. The structure of the Ka-band filter is shown in Figure 2, with design parameters highlighted in blue.

In this study, insertion loss (${\mathrm{dBS}}_{21}$) and the reflection coefficient (dB$S_{11}$) are used as the primary evaluation metrics for the filter model [10]. Insertion loss represents the signal transmission loss as it passes through the filter and is typically measured in decibels (dB). A lower insertion loss indicates better transmission performance, reducing signal reflection and power loss, thereby enhancing overall system performance—particularly in high-frequency applications.

The reflection coefficient characterizes the signal reflection at the filter’s input or output ports due to impedance mismatches. A lower reflection coefficient signifies better impedance matching, facilitating efficient signal transmission within the Ka-band and thereby minimizing signal loss [11].

**Figure 3:**
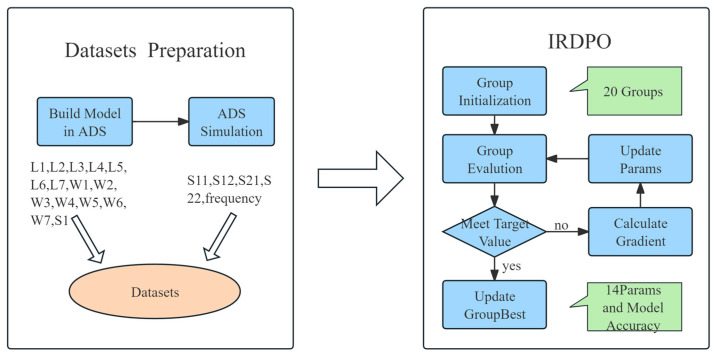
Optimization flow of the Ka-band filter using IRDPO.

This paper uses the improved RF device parameter extraction optimization algorithm (IRDPO) proposed in Section 2.4 to optimize the design parameters of the Ka-band filter. The process is shown in Figure 3 and consists of two main parts: the generation of the simulation dataset and the optimization search using the IRDPO algorithm.

When randomly generating the initial design parameters, this study selects an initial range of 100 µm to 2000 µm for the microstrip line length (L) and width (W). This range is determined based on previous design experience and practical manufacturing constraints, ensuring both design stability and integration feasibility.

To address the issue of irregular random distribution of design parameters in the dataset generation process, the Latin Hypercube Sampling (LHS) method is employed to generate a set of initial design parameter points with good coverage [12]. The LHS method enables uniform sampling across the design space, thereby enhancing the diversity and coverage of the simulation data. Using this approach, 20 sets of design parameters are generated to form the simulation dataset. IRDPO begins the optimization process from these initial design points, and multiple experiments are conducted to record the average results.

To achieve multi-objective optimization, the case study employs the Weighted Sum Method (WSM) to assign weights to the insertion loss (dB$S_{21}$) and reflection coefficient (dB$S_{11}$), integrating them into a single objective function. The objective function is defined as follows:

(12) $$F\left(s\right)=\omega_{1}\cdot MSE_{S21}+\omega_{2}\cdot MSE_{S11}$$

where $MSE_{S21}$ represents the Mean Squared Error (MSE) of the insertion loss:

(13) $$MSE_{S21}=\frac{1}{N}\overset{N}{\underset{i=1}{\sum }}{\left(dB_{S21}\left(s_{i}\right)-dB_{S21,target}\right)}^{2}$$

Similarly, $MSE_{S11}$ represents the MSE of the reflection coefficient:

(14) $$MSE_{S11}=\frac{1}{N}\overset{N}{\underset{i=1}{\sum }}{\left(dB_{S11}\left(s_{i}\right)-dB_{S11,target}\right)}^{2}$$

The MSE is used to measure the difference between the simulated values and the target values. A smaller MSE indicates that the predicted results of the model deviate less from the target values, leading to better optimization performance [13]. The coefficients $\omega_{1}$ and $\omega_{2}$ are the weights for insertion loss and the reflection coefficient, respectively, and they satisfy the following condition:$\omega_{1}+\omega_{2}=1$. The final optimization objective is to minimize $F\left(s\right)$ to achieve the combined optimization of the insertion loss and reflection coefficient.

## 4. Experimental Results

To validate the effectiveness of the improved RF device parameter extraction optimization algorithm proposed in this paper, Newton’s method (NM) [14], which is a deterministic optimization algorithm, Particle Swarm Optimization (PSO), and the Genetic Algorithm (GA) [15] are selected for comparison. To eliminate the influence of initial parameter values on the algorithm performance, multiple independent experiments were conducted for IRDPO, NM, PSO, and the GA. In each experiment, the initial parameter values were randomly generated, ensuring that the initialization range for all algorithms remained within 100 µm to 3000 µm.

**Figure 4:**
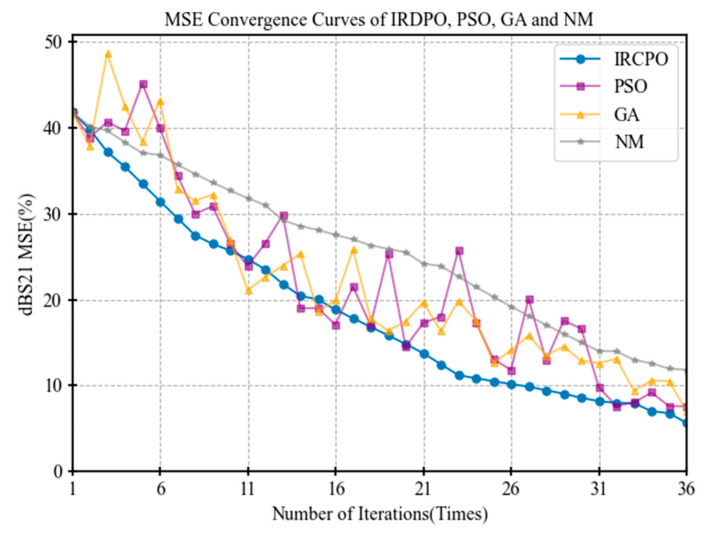
MSE convergence curve of dBS21 for IRDPO, PSO, GA, and NM.

In each experiment, the minimum error value in each iteration for a given initial parameter set was considered the optimal solution for that set, and the results were recorded accordingly. After multiple trials, the average value of the optimal solutions was computed, and an error convergence curve was plotted to objectively evaluate the optimization performance of each algorithm. The MSE convergence trend of the insertion loss ${\mathrm{dBS}}_{21}$ for the filter is illustrated in Figure 4.

From Figure 4, it can be observed that IRDPO exhibits significant advantages over the heuristic algorithms PSO and the GA during the optimization process. Specifically, the model error of IRDPO demonstrates a stable linear downward trend, indicating that it continuously improves model accuracy throughout the optimization process.

In the initial phase of optimization, the error reduction speed of IRDPO is significantly faster than that of PSO and the GA, reflecting its strong local search capability, which enables it to rapidly converge to high-quality solutions. Furthermore, multiple experimental results show that the IRDPO optimization results exhibit lower variability, with a smoother error convergence curve, demonstrating significantly higher stability compared to PSO and the GA.

This rapid convergence and high stability are particularly valuable in practical engineering applications, especially in scenarios where result consistency is crucial.

**Figure 5:**
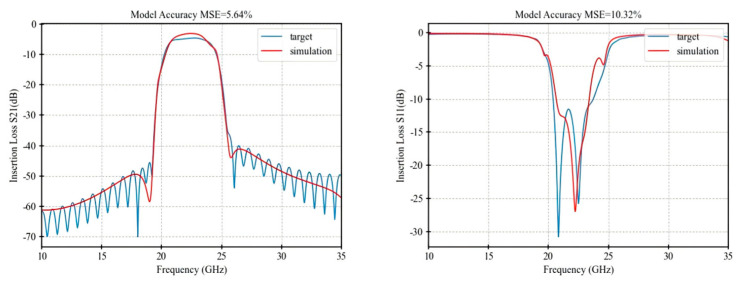
Curve of IRDPO.

**Figure 6:**
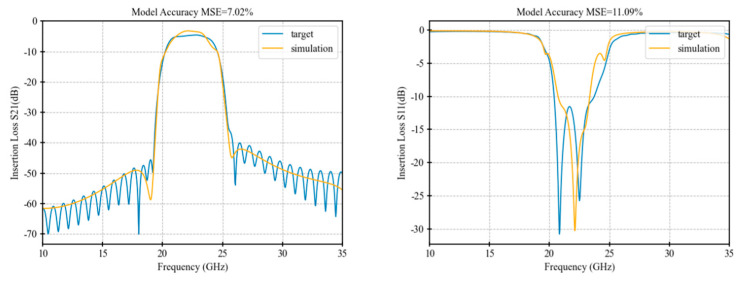
Fitting curve of GA.

**Figure 7:**
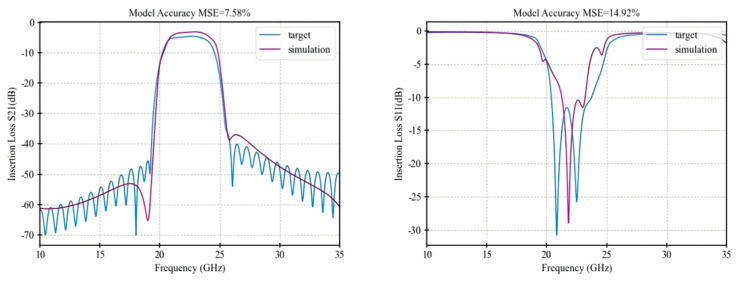
Fitting curve of PSO.

**Figure 8:**
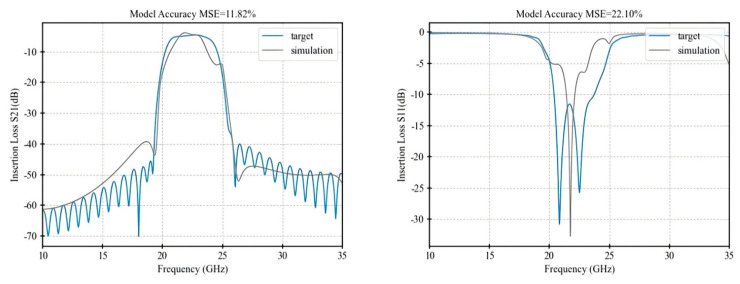
Fitting curve of NM.

Table 1 presents a performance comparison of the final optimization results among the three algorithms, while Figure 5, Figure 6, Figure 7 and Figure 8 illustrate the respective fitting curves for each algorithm.

Table 2 and Table 3 show the final optimized design parameters for IRDPO.

To further verify the performance of the four optimization algorithms under different error targets, this study uses the error between the target curve and the predicted curve of the reflection coefficient (dB$S_{11}$) as the optimization objective. The stopping criteria for the four optimization algorithms are set as follows: target errors of 15%, 10%, 8%, or reaching the maximum iteration count of 100. The experiments start from the same initial point, and parameters are optimized using IRDPO, NM, PSO, and the GA. The number of iterations required by each algorithm to achieve the target error is recorded.

To ensure the reliability of the results, multiple independent experiments are conducted. In each experiment, the initial parameter sets for the four algorithms are randomly generated within a reasonable range, and all algorithms run under the same initial conditions. The experimental results are shown in Table 4.

From Table 4, it can be observed that as the target error decreases, the number of iterations required by the four optimization algorithms gradually increases. IRDPO significantly requires fewer iterations than the GA and PSO at a 15% target error, reducing by 34.3% and 39.5%, respectively. At higher error targets, IRDPO achieves fast convergence due to its strong local convergence ability.

However, at the 8% target error, NM fails to converge. This does not mean that NM cannot achieve the target error, but rather that under the given maximum iteration limit (100 iterations), NM did not meet the convergence requirement. NM relies on second-order derivative information, which can lead to a relatively fast descent in the early stages. However, as the error target decreases, its step size gradually reduces, slowing down the optimization speed and requiring more iterations to complete the optimization. If more iterations were allowed, NM could eventually converge, but within the current 100 iteration limit, it did not meet the 8% error target. In contrast, IRDPO demonstrates higher computational efficiency among deterministic optimization algorithms, avoiding the computational bottleneck caused by the shrinking step size in NM and still efficiently converging to the target error within the limited iteration count.

On the other hand, the global search ability of the GA and PSO gradually compensates for their local convergence deficiencies at lower error targets, allowing them to perform similarly to IRDPO at an 8% target error. However, compared with the GA and PSO, IRDPO, as a deterministic optimization method, has a greater advantage in terms of optimization efficiency and stability. The GA and PSO rely on random searches, which, although strong in global exploration in complex search spaces, result in more variability in their optimization paths and require relatively more iterations due to the randomness. In contrast, IRDPO guides the search direction using gradient information, surpassing the GA and PSO in local convergence ability while maintaining a stable optimization path and avoiding the convergence fluctuations common in random algorithms.

Overall, IRDPO not only outperforms NM in deterministic optimization but also demonstrates faster convergence and more stable optimization performance than the random optimization algorithms, namely the GA and PSO. Its strong local optimization ability and stable parameter update mechanism make it excel in both optimization efficiency and accuracy, achieving the optimization target more effectively within a limited number of iterations.

## 5. Conclusions

This paper presents an improved RF device parameter extraction optimization algorithm (IRDPO), which significantly enhances optimization efficiency and convergence speed by incorporating gradient processing, bias-aware design parameter updates, and group optimization mechanisms. IRDPO demonstrates outstanding performance in Ka-band filter design parameter optimization, with experimental results showing that it outperforms Newton’s method, Particle Swarm Optimization (PSO), and the Genetic Algorithm (GA) in terms of convergence speed, local search capability, and result stability.

In this study, the Ka-band filter serves as a demonstration to validate the effectiveness of IRDPO. However, the application of the proposed algorithm is not limited to filter optimization. In fact, IRDPO can be widely applied to the design and optimization of other RF devices, especially for parameter extraction and performance optimization of RF transistors, such as High Electron Mobility Transistors (HEMTs), Field Effect Transistors (FETs), and Bipolar Junction Transistors (BJTs). Furthermore, IRDPO can also be applied to other RF circuits, such as power amplifiers, mixers, and antenna designs. These circuits typically involve complex parameter relationships and multi-objective optimization requirements. IRDPO can flexibly adjust objective functions and weight factors to precisely optimize circuit performance, ensuring the best balance under various design goals.

Future research can explore the integration of IRDPO with emerging technologies, such as deep learning, to improve its capabilities in high-dimensional and nonlinear optimization problems. This research will provide theoretical support and technological innovation for the field of Electronic Design Automation (EDA), contributing to the acceleration of intelligent RF circuit design and advancing the progress of next-generation RF technologies.

## Figures and Tables

**Table 1:** Accuracy comparison of optimization algorithms.

Performance	IRDPO	GA	PSO	NM
dBS21 MSE	5.64%	7.02%	7.58%	11.82%
Dbs11 MSE	10.32%	11.09%	14.92%	22.10%

**Table 2:** Length L of microstrip line.

Parameter	L1	L2	L3	L4	L5	L6	L7
Value(um)	1964.4	1615.0	2372.0	1790.5	2262.9	601.9	1357.0

**Table 3:** Width W and spacing S of microstrip line.

Parameter	W1	W2	W3	W4	W5	W6	W7	S1
Value(um)	499.3	305.7	284.1	202.0	240.4	604.1	121.8	7.9

**Table 4:** Iteration count comparison under different accuracy levels.

Target Error	IRDPO	GA	PSO	NM
15%	23	35	38	45
10%	42	49	55	92
8%	55	58	61	Not Converged

## Data Availability

The original contributions presented in this study are included in this article; further inquiries can be directed to the corresponding authors.
